# Temperamental and psychomotor predictors of ADHD symptoms in children born after a threatened preterm labour: a 6-year follow-up study

**DOI:** 10.1007/s00787-022-02073-9

**Published:** 2022-09-03

**Authors:** Pablo Navalón, Farah Ghosn, Maite Ferrín, Belén Almansa, Alba Moreno-Giménez, Laura Campos-Berga, Rosa Sahuquillo-Leal, Vicente Diago, Máximo Vento, Ana García-Blanco

**Affiliations:** 1 Neonatal Research Group, La Fe Health Research Institute, University and Polytechnic Hospital La Fe, Avenida Fernando Abril Martorell, 106, 46026 Valencia, Spain; 2 Division of Psychiatry and Clinical Psychology, La Fe University and Polytechnic Hospital, Valencia, Spain; 3 Department of Personality, Evaluation, and Psychological Treatments, Faculty of Psychology, University of Valencia, Valencia, Spain; 4 Haringey Children and Adolescent Mental Health Service, National Health Service, London, UK; 5 ReCognition Health, London, UK; 6 Division of Obstetrics and Gynecology, La Fe University and Polytechnic Hospital, Valencia, Spain; 7 Division of Neonatology, La Fe University and Polytechnic Hospital, Valencia, Spain

**Keywords:** ADHD, Neurodevelopmental disorders, Psychomotor development, Trauma, Pregnancy

## Abstract

Children born after threatened preterm labour (TPL), regardless of whether it ends in preterm birth, may represent an undescribed “ADHD cluster”. The aim of this cohort study is to identify early temperament and psychomotor manifestations and risk factors of TPL children who present ADHD symptoms. One hundred and seventeen mother–child pairs were followed from TPL diagnosis until the child’s 6 years of life. TPL children were divided according to the prematurity status into three groups: full-term TPL (*n* = 26), late-preterm TPL (*n* = 53), and very-preterm TPL (*n* = 38). A non-TPL group (*n* = 50) served as control. Temperament and psychomotor development at age 6 months and ADHD symptoms at age 6 years were assessed. Perinatal and psychosocial factors were also recorded. All TPL groups showed higher severity of ADHD symptoms compared with non-TPL children (difference in means + 4.19 for the full-term group, + 3.64 for the late-preterm group, and  + 4.99 for the very-preterm group, all *p*s < 0.021). Concretely, very-preterm and late-preterm TPL children showed higher restless/impulsive behaviours, whereas full-term TPL children showed higher emotional lability behaviours. Higher surgency/extraversion and delayed fine motor skills at age 6 months predicted ADHD symptoms at 6 years in TPL children. Male sex, maternal state anxiety symptoms at TPL diagnosis, low parental education, and past maternal experience of traumatic events predicted higher ADHD symptoms in TPL children. Therefore, TPL children may have a higher risk for developing ADHD symptoms, presenting a phenotype that depends on the prematurity status. Moreover, the specific combination of early manifestations and risk factors suggests that TPL children may conform an undescribed group at-risk of ADHD symptoms.

**Supplementary Information:**

The online version contains supplementary material available at 10.1007/s00787-022-02073-9.

## Introduction

Attention-Deficit/Hyperactivity Disorder (ADHD) is defined by persistent inattentive/disorganized and/or hyperactive/impulsive behaviours that lie at the end of a normally distributed continuum [1]. In addition to the genetic predisposition, current research has revealed the existence of perinatal and psychosocial risk factors during pregnancy that contribute to the aetiology of ADHD [2, 3]. It has been hypothesised that the different risk factors may be associated with a specific phenotype and with different severity levels, shaping different “ADHD clusters” [4]. Therefore, the main goal of this prospective study is to identify an undescribed ADHD cluster by focusing on a potential “at-risk of ADHD” population, in particular, children born after a threatened preterm labour (TPL).

TPL consists of experiencing regular and painful uterine contractions, together with cervical length changes that creates the possibility of giving birth prematurely [5]. It requires obstetric hospitalisation and may alter the normal course of pregnancy even in at-term children [6]. TPL diagnosis and treatment may include the exposure to several risk factors for ADHD. First, maternal anxiety after a TPL diagnosis can alter the hypothalamic–pituitary–adrenal axis (HPA) [7] and, thus, it may increase the risk of the child having symptoms of ADHD through an increased foetal exposure to cortisol [8]. Second, the treatment provided to women diagnosed with TPL (e.g., repeated courses of corticosteroids) may be related to hyperactivity symptoms in the offspring [9]. It has been found that infants born at term after a TPL have shown higher risk for impaired cognitive development [10]. Similarly, it has been demonstrated that, independent of prematurity, children born after a TPL have shown psychomotor and temperamental alterations [11], as well as higher autistic symptom load [12]. Hence, children born after a TPL may conform a population “at-risk” for developing another neurodevelopmental disorder such as ADHD.

To identify new potential clusters, follow-up studies have focused on the assessment of early temperamental and psychomotor manifestations of later ADHD symptoms [13]. Regarding temperamental manifestations, studies on the general population have found that higher extraversion, higher negative affectivity, and low emotional regulation in infants predicted later ADHD symptoms [14–18]. Considering psychomotor manifestations, deficits in gross motor and communication skills were predictors of later ADHD symptoms [19–22]. Among ADHD clusters, children born extremely preterm have been widely studied [23, 24]. The extremely preterm ADHD children cluster is characterized by a specific phenotype, consisting of higher proportion of the inattentive subtype [25, 26] and particular early manifestations (i.e., deficits in focused attention [27]; poor self-regulation [28]; and cognitive impairment [29]). Moreover, children born extremely preterm share specific risk factors, such as neonatal pain-related stress experiences [28]. Although previous research has reported better neurobehavioral outcomes with later delivery week [24, 30], recent studies point out that late preterm infants also show an increased risk for developing ADHD [25, 26]. These findings reinforce the idea that even at term children born after a TPL, regardless of the prematurity status, may conform an “ADHD cluster”.

Therefore, the aim of the current follow-up study is to examine: (i) the presence of ADHD symptoms at age 6 years in TPL children relative to non-TPL children, considering prematurity status; (ii) the association among psychomotor and temperament manifestations at age 6 months with ADHD symptoms at age 6 years in TPL children; and (iii) the presence of potential risk factors associated with perinatal (repeat doses of antenatal corticosteroids, gestational age at TPL diagnosis, gestational age at birth, birth weight percentile, multiple pregnancy, in vitro fertilisation, and sex) and psychosocial (maternal trait anxiety, maternal state anxiety and maternal cortisol levels at TPL diagnosis, social support, maternal experience of post-traumatic stress symptoms, parental education, and maternal and parental age) variables, and their association with ADHD symptoms at age 6 years in TPL children.

Based on the previous literature, we hypothesised that: (i) children born after TPL would show higher ADHD symptoms at age 6 years than non-TPL children [10], and that there is a phenotypic gradient, where the most severe presentation is observed on those extremely preterm children in comparison with other TPL groups [24, 30]; (ii) ADHD symptoms at age 6 years would be associated with specific early temperament and psychomotor manifestations at age 6 months in the TPL group [13, 19, 20, 28, 29]; and (iii) higher prenatal stress, in terms of higher maternal anxiety scores [7] and/or higher cortisol levels [31, 32], as well as treatment with repeated courses of corticosteroids [9], would be potential predictors for higher ADHD symptoms in TPL children.

## Method

### Participants

A prospective cohort study was conducted to follow pregnant women and the development of their offspring. A total sample of 167 mother–child pairs was recruited over a 1-year period that began in January 2015. Recruitment took place at the Obstetrics Unit of a tertiary hospital during the mother’s pregnancy. The mother–child pairs were divided into four groups after delivery, based on TPL diagnosis and week of gestation at birth. Children of mothers recruited after a TPL diagnosis were divided into the full-term TPL group (FT-TPL; *n* = 26) (born at or beyond 37 weeks of gestation), the late preterm TPL group (LP-TPL; *n* = 53) (born between 32 and < 37 weeks of gestation), and the very-preterm TPL group (VP-TPL; *n* = 38) (born with < 32 weeks of gestation). The non-TPL control group (*n* = 50) was composed of mothers and their full-term offspring (born at ≥ 37 weeks of gestation). The recruitment of control mothers was done between the 24th and 28th weeks of gestation during their glucose challenge test appointment, a routine screening for gestational diabetes done to all pregnant women. The children’s follow-up was carried out until the age of 6 years. The final sample was made up of mother–child pairs who participated in all the study assessment times (pregnancy, 6 months, and 6 years). This study was approved by the Research Ethical Committee of the institution. Informed consent was obtained from all the participants.

The inclusion criteria for the TPL groups were: (i) having regular and painful uterine contractions registered by cardiotocography; (ii) cervical length < 25 mm; and (iii) intact membranes. The TPL mothers’ recruitment was made between the 24th and 34th weeks of gestation in order to guarantee that they followed an identical protocol of tocolytic treatment [33]. Tocolytic treatment was implemented for at least 24 h. Additionally, TPL mothers received at least a single corticosteroid course (2 × 12 mg/24 h).

The exclusion criteria for all groups were: (i) history of major medical conditions that require chronic treatment and/or functional impairment (e.g., diabetes mellitus, high blood pressure, asthma, Body Mass Index < 17 or > 35, sexually transmitted infections, thyroid disease); (ii) severe obstetric complications; (iii) history of any psychiatric disorder, including ADHD, in both parents; (iv) social exclusion according to the Europe 2020 Strategy (i.e., at risk of poverty, severe material deprivation, or jobless households) [34]; (v) substance abuse/dependence and use of tobacco; or (vi) language barrier. Additionally, infants with congenital malformations, chromosomopathies, sensory impairments, severe postnatal diseases, or neurological disorders were also excluded.

### Assessment procedure

The evaluation was carried out at four time points. First, at TPL diagnosis or at the control’s first study appointment (between the 24th and 34th weeks of gestation), a socio-demographic semi-structured interview was conducted by trained psychologists, and an obstetrician reviewed the clinical history to collect obstetric details. At this point, participants provided a saliva sample (between 10:00 am and 12:00 pm, at least 1 h after breakfast) to determine cortisol levels and also completed scales related to anxiety (Spanish version of the State-Trait Anxiety Inventory, STAI; [35]), perceived social support (Multidimensional Scale of Perceived Social Support, MSPSS; [36]), and traumatic experiences (Trauma Questionnaire, TQ; [37, 38]). Second, after delivery, perinatal outcomes were recorded from the participants´ medical records. Third, temperament (Infant Behaviour Questionnaire-Revised Short Form, IBQ-R; [39]) and psychomotor development (Ages & Stages Questionnaires—Third Edition; ASQ-3; [40]) were assessed during the 6-month follow-up visit, when the infants were between the ages of 5 months 0 days and 6 months 30 days for at term infants and at corrected age for preterm infants. Finally, the last assessment took place 6 years after birth (between 69 months 0 days and 78 months 0 days of age). ADHD symptoms were evaluated at this time point using the Spanish version of the Conners Early Childhood Global Index (Conners ECGI) [41, 42]. All of the children’s assessments were conducted by two psychologists in the presence of at least one of the parents. Children’s abilities were directly observed to validate previous parental responses. See the Supplementary Material for the description of the assessment questionnaires and biological samples.

### Statistical analysis

Means and standard deviations were used for continuous variables, and the relative and absolute frequencies were used for categorical variables. The differences in the demographic characteristics and clinical data among FT-TPL, LP-TPL, VP-TPL, and non-TPL groups were examined using analysis of variance (ANOVA) for quantitative variables and the Chi-square statistic for categorical variables. Statistically significant differences between the groups on ADHD symptoms at age 6 years (Conners ECGI Total Score) were determined by conducting a one-way analysis of covariance (ANCOVA). In this analysis, multiple pregnancy was considered a confounder and it was included as a covariable. Then, the ADHD phenotypic presentation in TPL groups at age 6 years was examined conducting ANCOVAs to determine differences between groups regarding both Conners ECGI subscales (i.e., Restless/Impulsive and Emotional Lability). Again, multiple pregnancy was considered a confounder, and it was included as a covariable. Afterwards, two linear regression models were run only for the TPL groups. The first linear regression model aimed to predict ADHD symptoms at age 6 years in the TPL groups based on early 6-month psychomotor development scores (ASQ-3 Domains) and temperament scores (IBQ-R Factors). The second linear regression model aimed to examine the association of potential perinatal (repeat doses of antenatal corticosteroids, gestational age at TPL diagnosis, gestational age at birth, birth weight percentile, multiple pregnancy, in vitro fertilisation, and sex) and psychosocial (maternal trait anxiety, maternal state anxiety at TPL diagnosis, maternal cortisol levels at TPL diagnosis, social support, maternal experience of post-traumatic stress symptoms, parental education, and maternal and parental age) risk factors with ADHD symptoms at age 6 years in the TPL groups. The choice of predictive variables was carried out by a stepwise method, which was based on the *p* value of *F*, probability-to-enter (0.05) and probability to remove (0.10). A directed acyclic graph (DAG) was created to depict the associations among the different variables (i.e., perinatal and psychosocial predictors, early 6-month psychomotor development and temperament traits, and ADHD symptoms at age 6 years, generated by DAGitty v0.9b (available at http://www.dagitty.net/dags.html) (see Fig. S1 in the Supplementary Material) [43]. Finally, to observe if the early manifestations and risk factors of the TPL group are different from the control group, two additional linear regression models were performed for the non-TPL group (see Supplementary Material). The statistical analyses were performed using SPSS 25.0. The alpha level was set at *p* < 0.05 (two-tailed).

## Results

**Fig. 1:**
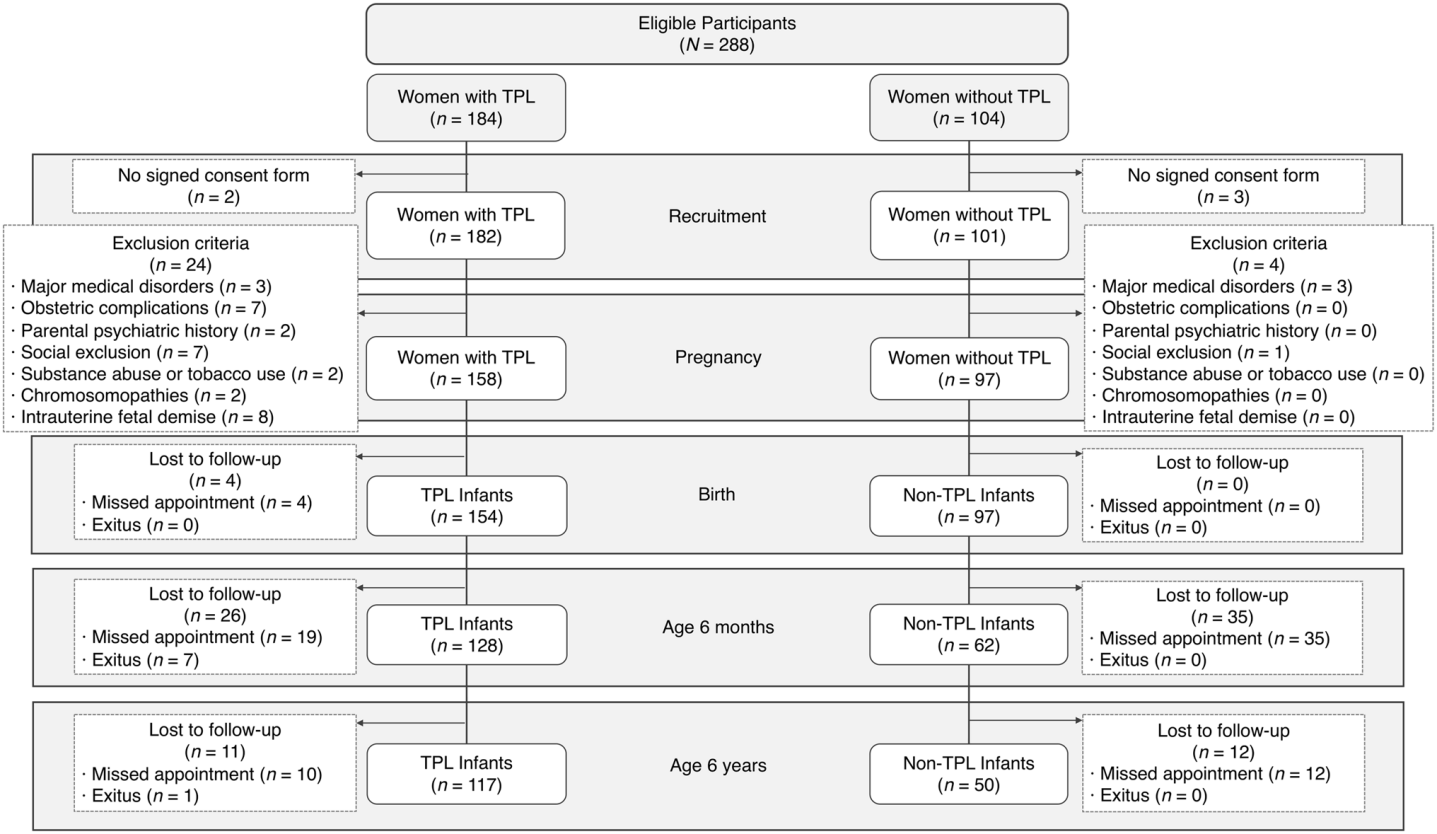
Recruitment flowchart

The final sample consisted of 117 TPL children and 50 non-TPL children. Figure 1 shows the flow diagram of the recruitment process and the sample losses. Participants that did not complete the full assessment at any time point were excluded from the analyses. Table 1 shows the demographic and clinical data. Table 2 shows the scores of ADHD symptoms at age 6 years and the psychomotor and temperament manifestations at age 6 months. Note that the groups showed significant differences on the number of participants with positive screening on the Conners ECGI at age 6 years: 32% for the very-preterm group, 27% for the full-term TPL group, 13% for the late-preterm group, and 4% for the control group.

**Table 1:** Demographic characteristics and clinical data of the sample

	Non-TPL (*n* = 50)	FT-TPL (*n* = 26)	LP-TPL (*n* = 53)	VP–TPL (*n* = 38)	*p*
Maternal age [*M* (*SD*)]	32.28 (4.21)	33.88 (4.27)	33.30 (4.97)	31.79 (4.81)	0.217
Paternal age [*M* (*SD*)]	35.68 (3.91)	35.92 (3.88)	35.75 (5.39)	35.89 (6.77)	0.997
Marital status					0.753
Cohabiting couples [*n* (%)]	42 (95%)	24 (96%)	49 (92%)	36 (97%)	
Separated/divorced [*n* (%)]	2 (5%)	1 (4%)	4 (8%)	1 (3%)	
Educational level					0.382
Primary [*n* (%)]	5 (11%)	7 (28%)	9 (17%)	11 (30%)	
Secondary [*n* (%)]	17 (37%)	8 (32%)	20 (38%)	13 (35%)	
University [*n* (%)]	24 (52%)	10 (40%)	24 (45%)	13 (35%)	
Gestations [*M* (*SD*)]	1.66 (0.80)	1.96 (1.54)	1.58 (.99)	1.53 (1.33)	0.454
Parity [*M* (*SD*)]	0.46 (0.58)	0.54 (1.14)	0.42 (0.72)	0.34 (.94)	0.804
Previous miscarriages [*M* (*SD*)]	0.20 (0.57)	0.42 (0.90)	0.17 (0.43)	0.21 (0.47)	0.303
IVF [*n* (%)]	3 (6%)	8 (31%)	15 (28%)	22 (58%)	** < 0.001**
Multiple pregnancy [*n* (%)]	0 (0%)	5 (19%)	33 (62%)	19 (50%)	** < 0.001**
Repeat doses of prenatal corticosteroids [*n* (%)]		10 (38%)	21 (40%)	11 (29%)	0.551
Gestational week at TPL [*M* (*SD*)]		29.88 (2.55)	30.62 (2.75)	27.20 (2.41)	** < 0.001**
Maternal cortisol levels (nmol L^−1^) [*M* (*SD*)]	4.92 (4.88)	1.75 (3.31)	3.30 (6.30)	4.26 (5.55)	0.084
STAI–T [*M* (*SD*)]	14.14 (6.81)	16.96 (7.83)	19.06 (9.76)	16.55 (9.14)	**0.041**
STAI–S [*M* (*SD*)]	12.73 (7.14)	19.88 (11.29)	20.62 (8.64)	24.00 (11.06)	** < 0.001**
MSPSS [*M* (*SD*)]	79.40 (6.25)	79.96 (4.49)	79.06 (5.69)	78.55 (6.48)	0.807
TQ [*M* (*SD*)]	1.78 (2.99)	2.85 (4.38)	2.34 (3.33)	54.84 (5.50)	**0.004**
Weight at birth (g) [*M* (*SD*)]	3279.70 (424.73)	3065.21 (412.99)	2093.40 (424.84)	1205.14 (453.80)	** < 0.001**
Birth weight percentile for gestational age [*M* (*SD*)]	45.72 (29.00)	44.12 (27.01)	35.25 (27.93)	48.03 (32.15)	0.153
Delivery week [*M* (*SD*)]	39.89 (.86)	38.46 (1.31)	34.50 (1.44)	28.62 (2.43)	** < 0.001**
Male sex [*n* (%)]	25 (50%)	13 (50%)	32 (60%)	20 (53%)	0.709
Apgar 1 [*M* (*SD*)]	8.88 (1.77)	9.32 (.67)	8.65 (1.37)	6.87 (2.15)	** < 0.001**
Apgar 5 [*M* (*SD*)]	9.85 (.42)	9.89 (.32)	9.81 (.49)	8.55 (1.55)	** < 0.001**
NICU admission [*n* (%)]	0 (0%)	0 (0%)	10 (19%)	30 (79%)	** < 0.001**
Neonatal hospital admission [*n* (%)]	6 (12%)	2 (10%)	40 (78%)	37 (100%)	** < 0.001**
Feeding					0.319
Formula milk [*n* (%)]	11 (22%)	5 (25%)	18 (35%)	10 (27%)	
Breast milk [*n* (%)]	39 (78%)	13 (65%)	31 (61%)	25 (68%)	
Mixed [*n* (%)]	0 (0%)	2 (10%)	2 (4%)	2 (5%)	

The *p* values correspond to Chi-squared tests for qualitative variables and to *t* tests for quantitative variables

*Non-TPL* children born at full term without a threatened preterm labour; *FT-TPL* children born at full term after a threatened preterm labour; *LT-TPL* late-preterm children born after a threatened preterm labour; *VP-TPL* very-preterm children born after a threatened preterm labour; *IVF* in vitro fertilisation; *TPL* threatened preterm labour; *STAI-T* state-trait anxiety inventory-trait form; *STAI-S* state-trait anxiety inventory-state form; *MSPSS* Multidimensional Scale of Perceived Social Support; *TQ* Traumatic Experiences Questionnaire; *NICU* neonatal intensive care unit

*p* values < 0.05 are shown in bold

**Table 2:** Scores of non-TPL children and TPL children on ADHD symptoms, psychomotor development, and temperament questionnaires

	Non-TPL (*n* = 50)	FT-TPL (*n* = 26)	LP-TPL (*n* = 53)	VP-TPL (*n* = 38)	*p*
Total score on Conners ECGI at age 6 years [*M* (*SD*)]	7.24 (4.25)	11.27 (5.14)	10.36 (5.46)	11.82 (6.23)	** < 0.001**
Restless impulsive [*M* (*SD*)]	5.62 (3.24)	8.15 (3.76)	7.87 (4.44)	8.92 (4.69)	** < 0.001**
Emotional lability [*M* (*SD*)]	1.76 (1.61)	3.12 (2.01)	2.89 (2.06)	2.89 (2.04)	**0.005**
Positive screening [*n*(%)]^a^	2 (4%)	7 (27%)	7 (13%)	12 (32%)	**0.003**
ASQ-3					
Communication skills [*M* (*SD*)]	55.80 (7.72)	48.27 (13.63)	48.87 (12.92)	44.87 (17.49)	** < 0.001**
Gross motor skills [*M* (*SD*)]	52.00 (7.69)	51.73 (10.67)	48.87 (9.84)	42.76 (15.36)	** < 0.001**
Fine motor skills [*M* (*SD*)]	52.30 (7.09)	48.46 (13.91)	44.06 (13.62)	39.21 (17.80)	** < 0.001**
Problem solving skills [*M* (*SD*)]	55.90 (5.95)	51.15 (9.31)	48.02 (11.11)	41.71 (17.17)	** < 0.001**
Personal social skills [*M* (*SD*)]	58.40 (4.45)	52.88 (9.51)	51.04 (11.11)	44.08 (16.10)	** < 0.001**
IBQ-R					
Negative affectivity [*M* (*SD*)]	2.84 (0.82)	3.31 (0.65)	3.41 (0.58)	3.12 (0.75)	** < 0.001**
Surgency/extraversion [*M* (*SD*)]	4.59 (0.60)	4.39 (0.86)	4.29 (0.58)	3.93 (0.84)	** < .001**
Orienting/regulation [*M* (*SD*)]	5.51 (0.70)	4.59 (0.68)	4.68 (0.73)	4.62 (0.61)	** < 0.001**

*Non-TPL* children born at full term without a threatened preterm labour; *FT-TPL* children born at full term after a threatened preterm labour; *LT-TPL* late-preterm children born after a threatened preterm labour; *VP-TPL* very-preterm children born after a threatened preterm labour; *Conners ECGI* Conners Early Children Global Index; *ASQ-3* Ages and Stages Questionnaire-Third Edition; *IBQ-R* Infant Behaviour Questionnaire-Revised

*p *values < 0.05 are shown in bold

^a^Values reflect the number and percentage of children with a positive screening on the Conners ECGI [total score cut-off points (*T* ≥ 70) = 13 (female) and 15 (male)]

### ADHD symptoms at age 6 years

#### Are ADHD symptoms of TPL children different from that of non-TPL children at age 6 years?

The ANCOVA revealed a main effect of Group [*F*(3, 162) = 6.40, *p* < 0.001, η_p_^2^ = 0.106] after controlling for multiple pregnancies. Bonferroni comparisons indicated that the FT-TPL (Difference in Means (DM) =  + 4.19, *d* = 0.854*, p* = 0.009), the LP-TPL (DM =  + 3.64, *d* = 0.637, *p* = 0.021), and the VP-TPL (DM =  + 4.99, *d* = 0.858, *p* = 0.001) groups showed higher ADHD symptoms than the non-TPL group. No other between-group differences were found (all *p*s = 1).

#### ADHD phenotype in TPL children at age 6 years

**Fig. 2:**
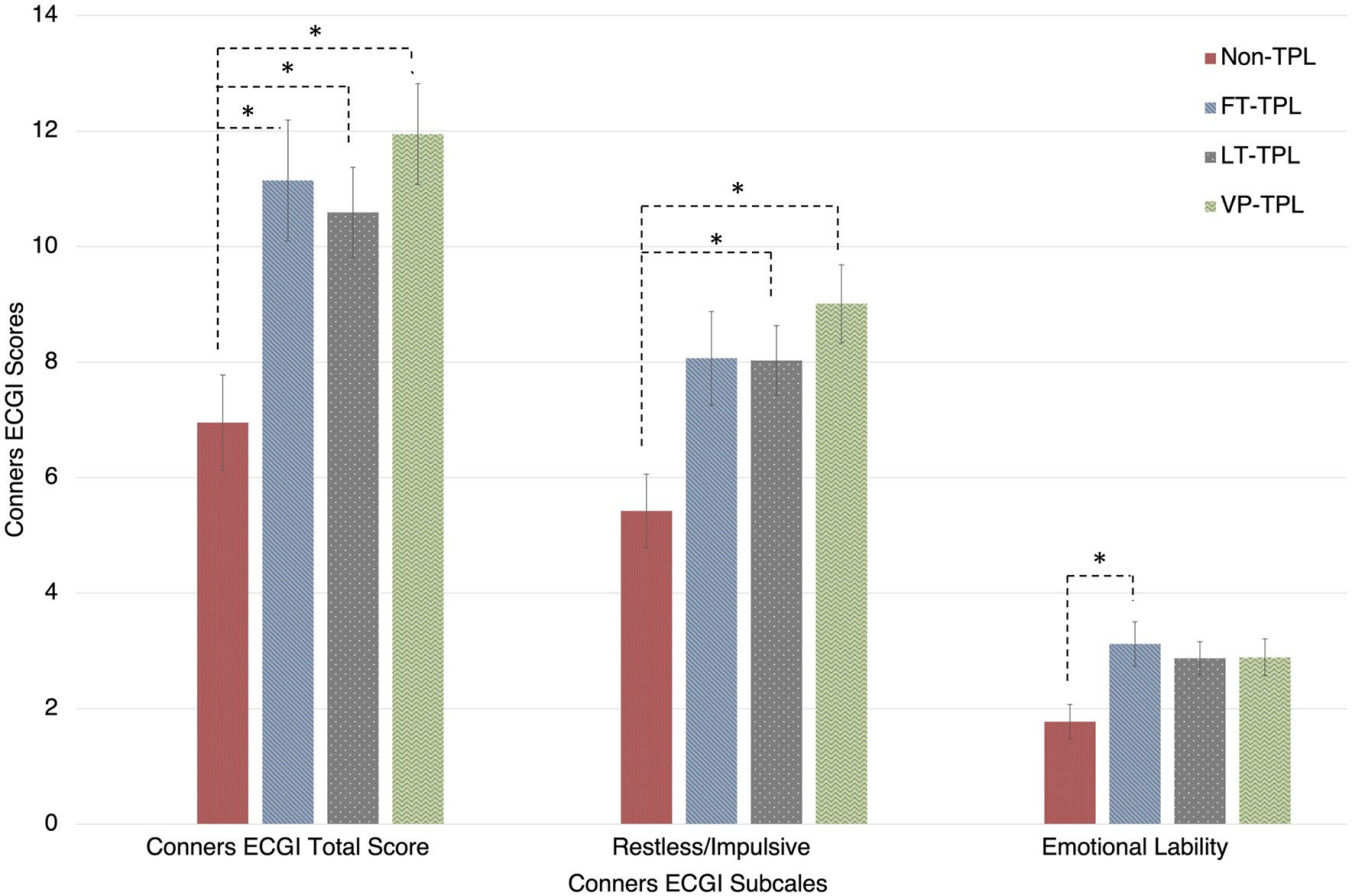
ADHD symptoms and phenotypic presentation in non-TPL children, full-term TPL children, late preterm TPL children, and extremely preterm TPL children at age 6 years. * means *p* < 0.05

After controlling for multiple pregnancies, the ANCOVAs revealed a significant effect of Group on Restless/Impulsive [*F*(3, 162) = 5.17, *p* = 0.002, *η*_*p*_^2^ = 0.087] and on Emotional lability [*F*(3, 162) = 3.56, *p* = 0.016, *η*_*p*_^2^ = 0.062]. Bonferroni comparisons revealed that the VP-TPL (*d* = 0.819, *p* = 0.002) and LP-TPL (*d* = 0.578, *p* = 0.039) groups obtained higher scores in the Restless/Impulsive subscale than the non-TPL group. On the other hand, the FT-TPL group (*d* = 0.745, *p* = 0.029) showed greater scores in the Emotional Lability subscale than the control group. No other between-groups comparisons achieved significance (all *p*s > 0.053) (see Fig. 2).

#### Early temperamental and psychomotor manifestations as predictors of ADHD symptoms at age 6 years in TPL children

The predicted amount of variance in the Conners ECGI total score at age 6 years in TPL infants was 10.15–0.15 (Fine Motor Skills) + 1.63 (Surgency/Extraversion), [*F*(2, 119) = 11.34, *p* < 0.001, *R*^2^ = 0.16, *R*^2^_Adjusted_ = 0.15]. Thus, delays in Fine Motor Skills development and higher Surgency/Extraversion at age 6 months predicted an increase of ADHD symptoms at age 6 years in TPL children.

#### Potential risk factors of ADHD symptoms at age 6 years in TPL children

The predicted amount of variance in the Conners ECGI Total Scores at age 6 years in TPL infants was 12.17–3.98 (Sex) + 0.15 (Maternal State Anxiety) + 0.29 (Maternal Experience of Post-Traumatic Stress Symptoms) -1.74 (Parental Education), [*F*(4, 107) = 10.02, *p* < 0.001, *R*^2^ = 0.27, *R*^2^_Adjusted_ = 0.25]. Thus, being male, having high levels of maternal state anxiety at TPL diagnosis, having a mother who has experienced post-traumatic stress symptoms, and low parental education at TPL diagnosis were the main predictors of higher severity of ADHD symptoms at the age of 6 years in TPL children.

## Discussion

The results support that children born after a TPL, regardless of the prematurity status, conform a novel at-risk population for developing ADHD symptoms, with a particular phenotype and a specific combination of associated risk factors and early temperamental and psychomotor manifestations. Concretely, it was found that: (a) compared with non-TPL children, all TPL children groups showed higher ADHD symptoms at 6 years of age; (b) very-preterm and late-preterm TPL children showed higher restless/impulsive symptoms, whereas full-term TPL children showed higher emotional lability at age 6 years compared to non-TPL infants; (c) delays in fine motor skills and higher extraversion at age 6 months predicted greater ADHD symptoms at age 6 years in TPL children; and (d) male sex, having high levels of maternal state anxiety at TPL diagnosis, having a mother who has experienced post-traumatic stress symptoms, and having parents with a low educational level were identified as the main predictors of increased ADHD symptoms at age 6 years in TPL children. Altogether, the findings suggest that TPL infants may conform an undescribed ADHD cluster.

Regarding ADHD symptoms, children born after a TPL showed higher Conners ECGI scores at age 6 years compared to non-TPL children, although these symptoms did not reach, on average, the clinical threshold. Nevertheless, considering the Conners ECGI cut-off points, the risk for ADHD increased between 3- and 8-fold in children born after a TPL. This finding suggests that an episode of TPL may be a key point involved in the deleterious effect of prematurity on neurodevelopment, and it may alter the course of pregnancy even in at-term children [6]. Similarly, other studies have found that TPL is involved in impaired neurodevelopmental trajectories, even if birth occurred at term [10–12]. In addition, unlike previous studies [30], we did not find higher symptomatic severity in very-preterm children compared to the rest of TPL children. Conversely, we found a phenotypic ADHD gradient rather than a severity ADHD gradient. In our sample, very-preterm and late-preterm TPL children had higher symptoms related to restlessness/impulsivity, whereas full-term TPL children showed higher emotional lability traits, compared to non-TPL children. It is important to note that the restless/impulsive subscale includes inattentive symptoms. In this line, preterm children have been found to show higher inattentive symptoms also at school age [44, 45]. Notwithstanding, as far as we know, this is the first study that has specifically analysed the differences in the phenotypic manifestations based on the prematurity status.

Considering early psychomotor and temperament predictors of ADHD symptoms, the results indicate that delayed fine motor skills development and increased extraversion at age 6 months predicted ADHD symptoms at age 6 years in TPL children. Similarly, extraversion/surgency traits have been identified as predictors of ADHD symptoms in the general population [14, 15]. Moreover, a delay in psychomotor development is also predictive of later ADHD diagnosis in the general population [20, 22]. However, the TPL groups did not show other early psychomotor and temperament signs previously identified in the general population such as high negative affectivity impairments, low emotional regulation, or gross motor alterations [19, 22]. Finally, the early predictors of ADHD symptoms in the TPL groups were also different from the ones of very-preterm children previously identified (attentional, self-regulation, and cognitive impairments [27–29]). Thus, the findings suggest a specific combination of early temperament and psychomotor signs in TPL children. Although the significant associations among psychomotor and temperament alterations at age 6 months with ADHD symptoms at age 6 years may suggest a specific clinical trajectory of ADHD in TPL children, these observed early life psychomotor and temperament manifestations overlap with those of other early childhood mental disorders [46–49].

Finally, regarding risk factors, it was found that male sex, having high levels of maternal state anxiety at TPL diagnosis, having a mother who has experienced post-traumatic stress symptoms, and parent’s low educational level were the main predictors of higher ADHD symptoms at age 6 years in TPL children. Male sex and parent’s low educational level have been identified as ADHD risk factors in the general population [3, 20, 50]. Contrarily, other previously identified risk factors for ADHD, such as preterm birth, low birth weight, advanced parental age, IVF, or low social support, have not been found to be significant predictors of ADHD symptoms in TPL infants. We initially hypothesised that the treatment with repeated courses of corticosteroids [9] could be the aetiological mechanism explaining ADHD symptoms in TPL children; however, this hypothesis was not supported by the results. A plausible explanation is that repeated courses of corticosteroids may increase the risk of neurodevelopmental alterations not necessarily associated with ADHD but rather to other neurodevelopmental areas. Interestingly, we found that both having high levels of maternal state anxiety at TPL diagnosis and having a mother who has experienced post-traumatic stress symptoms predicted ADHD symptoms in children born after a TPL. This highlights the importance of both prenatal stress and previous traumatic events for pregnant women [51]. Previous studies have shown that TPL diagnosis is a stressful prenatal event that may have an impact on the foetal neurodevelopment [31, 32, 52]. Thus, high cortisol levels were expected to show an association with higher severity of ADHD symptoms. However, the results did not show any association in this study. A possible explanation is that cortisol levels rise in all pregnant women as the gestation progresses and its levels significantly fluctuate throughout the day [53]. Therefore, measuring cortisol levels during pregnancy may not provide a reliable stress measurement compared to the maternal state anxiety questionnaire. As for pregnant women who has experienced post-traumatic stress symptoms, research on traumatic events reveals that they could increase the risk for experiencing subsequent traumas [54]. Thus, we propose that experiencing complications during pregnancy, such as TPL in women who have had past experiences of traumatic events, could be a form of revictimization, leading to experiencing the situation as traumatic [55]. Moreover, lower educational level also predicts post-traumatic stress symptoms in women suffering from obstetric complications [56]. Furthermore, it has been found that maternal traumatic experiences are associated with hormonal and epigenetic alterations in the offspring [57, 58], which eventually may be associated with altered infant neurodevelopment [59]. In sum, these findings support that those children born after a TPL share a specific combination of psychosocial risk factors related to maternal anxiety symptoms, past post-traumatic stress experiences, and low educational level, in addition to male sex. In turn, these psychosocial factors are associated with lower levels of personal resources, economic difficulties, self-care deficits, insufficient access to antenatal care, isolation, and/or discrimination [51], and may be related to potential aetiological mechanisms of ADHD in TPL children, which may become an important area of research.

The present study has several strengths. First, assessments were made by both parents and psychologists, increasing the reliability. Second, using the Conners ECGI led us to consider ADHD symptoms instead of ADHD diagnosis, giving us the opportunity to examine the disorder from a dimensional perspective. Third, this study represents one of the few prospective studies that start during the mothers’ pregnancies. However, there are some limitations. First, the strict inclusion/exclusion criteria prevented us to get a larger sample size, which could have resulted in a higher statistical power to find more early manifestations and predictors, especially in the control group. Second, although the STAI questionnaire and salivary cortisol are useful tools widely employed in research, they may be low sensible measurements to assess stress in hospitalised pregnant mothers. Third, although parents with ADHD diagnosis were excluded, we did not measure subsyndromal ADHD symptoms, which could be associated with ADHD traits in the offspring. Fourth, 74.1% of TPL children completed the follow-up study, whereas 51.5% of the control group were followed up until the age of 6 years. This difference may be explained by a lower parental concern about their children’s development, since they did not suffer from perinatal alterations. Finally, although strict selection criteria were chosen, we cannot reject the contribution of unmeasured genetic and familiar factors (e.g., quality of mother–child relationship) that could have had an effect on the child’s development.

## Conclusions

This study suggests that TPL children, even those who are born at term, may conform an undescribed “at-risk” population for showing ADHD symptoms. Similar to previously identified ADHD clusters, TPL children show a particular combination of early signs, such as having poor fine motor skills and greater surgency/extraversion at 6 months of age. TPL children also present shared risk factors, namely male sex, having high levels of maternal state anxiety at TPL diagnosis, having a mother who has experienced post-traumatic stress symptoms, and low parental education. Critically, the ADHD phenotype of TPL children depends on the prematurity status. Therefore, these findings suggest that TPL children may be a population “at-risk” of ADHD symptoms, with shared risk factors and with specific clinical trajectories. Further research in this area is needed to explain the aetiological mechanisms of ADHD in this population.

### Supplementary Information

Below is the link to the electronic supplementary material.

Supplementary file1 (DOCX 97 kb)

## Data Availability

Due to the sensitive nature of the questions asked in this study, participants were assured that their individual data would remain confidential and would not be shared. Data related to the measuring tools can be found in the tables.
