# Supplementary material for: Temperamental and psychomotor predictors of ADHD symptoms in children born after a threatened preterm labour: a 6-year follow-up study
Source: Eur Child Adolesc Psychiatry. 2022 Sep 3;32(11):2291–301. doi: 10.1007/s00787-022-02073-9 (PMC10576661; doi:10.1007/s00787-022-02073-9)

**Supplementary Material**

**Methods**

***Sample Size Calculation***

The sample size was determined based on statistical power, effect size, time, and funding. A total number of 185 TPL pregnant women recruited during a one-year period was a realistic and achievable sample size in our clinical setting. Assuming a loss to follow-up of around 20% and case exclusion of around 10%, 130 cases would complete the six-year follow-up visit, of which 30% will be born at <32 weeks of gestation and 70% at ≥32 weeks of gestation. Therefore, given that 40% of very preterm children and 20% of late preterm children have a positive ADHD screening at six years of age, it is estimated that at least 34 TPL children of our sample would exhibit ADHD symptoms. For an alpha value (type I error) of 5% and a statistical power (1 – beta) of 80%, we estimated that our sample should be sufficient to obtain statistically significant results.

***Description of the Assessment Questionnaires and Biological Sample***

**Maternal Psychological Assessment.** The Spanish version of the State-Trait Anxiety Inventory (STAI) [1] was administered to all mothers at the recruitment moment. The STAI is a well-known inventory used in different populations to determine two types of anxiety, i.e., trait anxiety and state anxiety. The STAI has two forms, designed to either measure trait anxiety (STAI-T) or state anxiety (STAI-S). Each form is composed of 20 items that are rated on a 4-point Likert scale (almost never = 0, sometimes = 1, often = 2, and almost always = 3). Total scores can range from 0 to 60 for each form. Raw scores above 24 for trait anxiety and above 21 for state anxiety (above the 50^th^ percentile) indicate moderate trait or state anxiety in females. Higher scores indicate higher severity of trait or state anxiety. In this study, trait anxiety and state anxiety were measured as predictive variables of maternal anxiety at TPL diagnosis/glucose challenge test.

**Assessment of Perceived Social Support.** To evaluate the mothers´ perception of social support, the Multidimensional Scale of Perceived Social Support (MSPSS) [2] was used at the recruitment moment. It is a brief self-reported tool with 12-items divided into three subscales, each addressing a different source of support: (a) Family (e.g., “*My family really tries to help me*”), (b) Friends (e.g., “*I can count on my friends when things go wrong*”), and (c) Significant Other (e.g., “*There is a special person in my life who cares about my feelings*”). Each item is rated on a 7-point Likert-scale ranging from *very strongly disagree* (1) to *very strongly agree* (7). MSPSS was found to have good internal and test-retest reliability, and a strong construct validity. Total raw scores can range from 12 to 84 and indicate the degree of perceived social support [low (0-35), moderate (36-60), and high (61-84)]. Thus, high scores indicate high levels of perceived social support.

**Assessment of Traumatic Experiences.** The Trauma Questionnaire (TQ) is a simple self-administered tool created to screen for posttraumatic stress disorder (PTSD) and used to measure traumatic events [3, 4]. The TQ was answered by all mothers at the recruitment moment. It is divided into three well-differentiated parts: a) list of traumatic experiences quantifying the life history of stressful events and their duration (19 items) b) characteristics of the most upsetting traumatic event (9 items), and c) list of PTSD symptoms associated with said event (18 items). Items are dichotomously scored (yes = 1, no = 0) and only the last 18 items are considered when calculating the total score. Thus, the total score ranges from 0 to 18, with higher scores indicating higher severity of PTSD symptoms.

**Assessment of Psychomotor Developmental Milestones.** The Ages & Stages Questionnaires - Third Edition (ASQ-3) [5] are a developmental screening tool that identifies the developmental progress in children aged from 1 month to 5.5 years. Specifically, the 6-month questionnaire (designed to assess children aged between 5 months 0 days and 6 months 30 days) was applied to infants during the 6-month assessment visit. The questionnaire screens five different domains: communication skills, gross motor skills, fine motor skills, problem-solving skills, and personal-social skills. The parents rated whether each item applied to their infant by responding “yes”, “sometimes”, or “not yet” to the six items conforming each domain. Parents’ responses were revised by two clinical psychologists to guarantee that they were reliable. Every answer has a fixed score (“yes” = 10, “sometimes” = 5, and “not yet” = 0). Total scores range from 0 to 60 for each psychomotor domain, with high scores indicating that the infant’s development progresses appropriately.

**Temperament Measuring Tool.** The Infant Behaviour Questionnaire-Revised (IBQ-R) Short Form [6] is a widely used tool to assess temperament in infants aged between 3 and 12 months. Parents responded to this questionnaire during the 6-month assessment visit. The tool was administered by a clinical psychologist to ensure that parents understood the items and that the responses were coherent with the clinical observation. The questionnaire measures three major temperament factors: i) surgency/extraversion; ii) negative affectivity; and iii) orienting/regulation*.* The IBQ-R has 91 items, each rated using a 7-point Likert-scale (*never* = 1, *very rarely* = 2, *less than half the time* = 3, *about half the time* = 4, *more than half the time* = 5, *almost always* = 6, *always* = 7, and *does not apply* = NA). Higher scores indicate higher levels of each temperament factor.

**Assessment of ADHD Symptoms.** To evaluate ADHD symptoms at age six years, the Spanish version of the Conners ECGI [7] was employed. The Conners ECGI [8] is used to assess the presence of developmental delays or general psychopathology in children between the ages of 2 and 6 years. It measures the presence of ADHD symptoms in two subscales: i) restless/impulsive (i.e., distractibility, restlessness, fidgety, or impulsivity) and ii) emotional lability (i.e., crying, frustration, outbursts, and mood changes). It is composed of 10 items that, in this study, were responded by the parents. Each item is scored on a four-point Likert scale that ranges from *not true at all* (0) to *very much true* (3). Total scores can range from 0 to 30, and the higher the score the more concerning the symptoms. The scale has cut-off points for elevated scores (T ≥65; 12 points for female and 14 for male) and very elevated scores (T ≥70; 13 points for female and 15 for male), indicating more or many more concerning symptoms, respectively. The parents’ form has shown good internal reliability and inter-rater agreement, and it is considered a valid instrument for detecting ADHD symptoms [7]. It was also administered to parents by a clinical psychologist to ensure that they understood the items and that the responses were coherent with the clinical observation.

**Biological Measure of Maternal Physiological Stress.** In order to analyse salivary cortisol as a stress biomarker during pregnancy, a saliva sample was collected from all the mothers using saliva collection tubes at the recruitment moment. This procedure was conducted between 10:00 am and 12:00 pm, at least 1 hr after breakfast. Before the analysis, the saliva samples remained in a refrigerator at -80ºC. Later, they were thawed and homogenized for the analysis. To extract the salivary cortisol, 25 μL of each sample was subjected to liquid-liquid extraction, and the residues were reconstituted in a water [0.1% (v/v) HCOOH, pH 3]: methanol (85:15 v/v) solution. Then, 5 μL of the solution was injected in an ultra-performance liquid chromatograph coupled to tandem mass spectrometer (Waters Acquity UPLC-Xevo TQD system, Milford, MA, USA). These analyses were based on a previous study conducted by ﻿García-Blanco et al. [9]. The intra- and inter-day coefficients of variation in these analyses were 12% (*n* = 3) and 13% (*n* = 9), respectively (at a concentration of 20 nmol L^−1^). The obtained limit of detection and limit of quantification for cortisol were 0.05 nmol L^–1^ and 0.1 nmol L^−1^, respectively. The cortisol standards were purchased from Sigma-Aldrich (Madrid, Spain).

**Results**

***Early Temperamental and Psychomotor Manifestations as Predictors of ADHD Symptoms at Age Six Years in Non-TPL Children***

Temperament and psychomotor manifestations at age six months were assessed as predictors of ADHD symptoms at age six years in non-TPL children. The predicted amount of variance in the Conners ECGI Total Scores at age six years in non-TPL children was - 8.12 + 2.81 (Orienting/Regulation), [*F*(1, 50) = 13.46, *p* = .001, *R*^2^ = .212, *R*^2^_Adjusted_ = .196]. Thus, poor Orientating/Regulation capacity at age six months predicted an increase of ADHD symptoms at age six years in the non-TPL children.

***Potential Risk Factors of ADHD Symptoms at Age Six Years in Non-TPL Children***

The variables related to potential ADHD risk factors (gestational age at birth, birth weight percentile, multiple pregnancy, *in vitro* fertilisation, sex, maternal trait anxiety, maternal state anxiety and maternal cortisol levels at recruitment, social support, maternal experience of post-traumatic stress symptoms, parental education, and maternal and parental age) were assessed as predictors of ADHD symptoms at age six years. None of the potential ADHD risk factors were found to be statistically significant predictors of Conners ECGI Total Scores at age six years in non-TPL children.

**References**

1. Spielberger RD, Gorsuch RL, Lushene RE (1982) Manual STAI, Cuestionario de Ansiedad Estado Rasgo. Tea Ediciones 23:3–14

2. Zimet GD, Dahlem NW, Zimet SG, Farley GK (1988) The Multidimensional Scale of Perceived Social Support. J Pers Assess 52:30–41. https://doi.org/10.1207/s15327752jpa5201_2

3. Bobes J, Calcedo-Barba A, García M, et al (2000) [Evaluation of the psychometric properties of the Spanish version of 5 questionnaires for the evaluation of post-traumatic stress syndrome]. Actas Esp Psiquiatr 28:207–18

4. Davidson J, Smith R (1990) Traumatic experiences in psychiatric outpatients. J Trauma Stress 1990 33 3:459–475. https://doi.org/10.1007/BF00974785

5. Squires J, Bricker D (2009) Ages & Stages Questionnaires (3rd edition): user’s guide

6. Putnam SP, Helbig AL, Gartstein MA, et al (2014) Development and assessment of short and very short forms of the infant behavior questionnaire-revised. J Pers Assess 96:445–458. https://doi.org/10.1080/00223891.2013.841171

7. Morales-Hidalgo P, Hernández-Martínez C, Vera M, et al (2017) Psychometric properties of the Conners-3 and Conners Early Childhood Indexes in a Spanish school population. Int J Clin Health Psychol 17:85–96. https://doi.org/10.1016/j.ijchp.2016.07.003

8. Conners C, Goldstein S (2009) Conners early childhood: Manual

9. García-Blanco A, Vento M, Diago V, Cháfer-Pericás C (2016) Reference ranges for cortisol and α-amylase in mother and newborn saliva samples at different perinatal and postnatal periods. J Chromatogr B 1022:249–255. https://doi.org/10.1016/j.jchromb.2016.04.035

**Figure S1.** Directed acyclic graph (DAG) created to assess the links among the different variables.


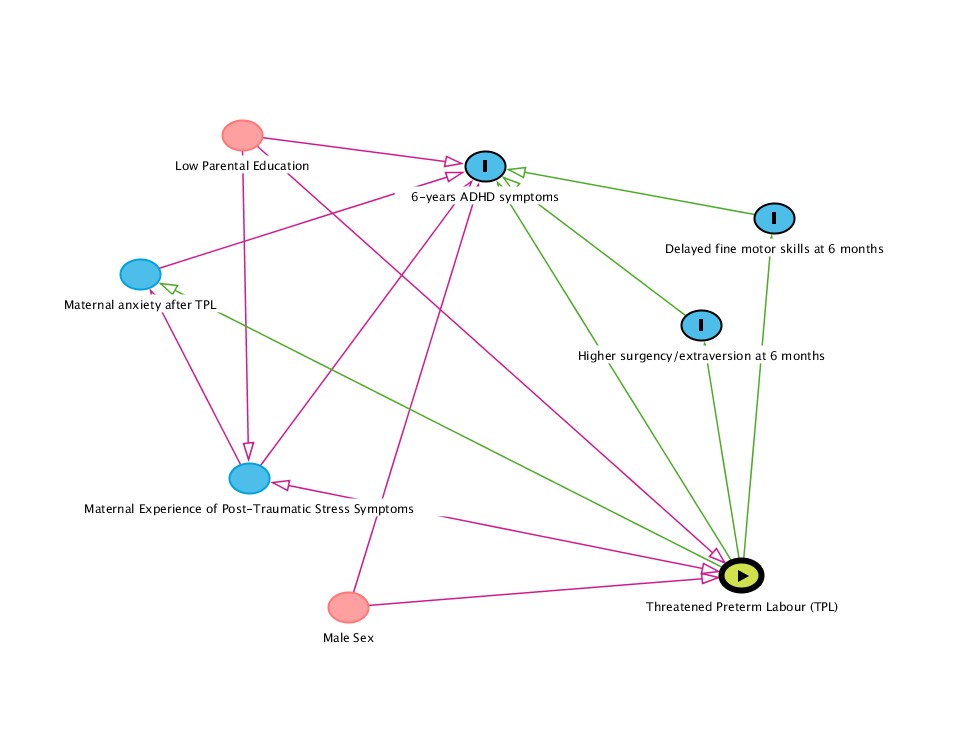

Supplement: Supplementary file 1 — Supplementary file1 (DOCX 97 kb) [file 787_2022_2073_MOESM1_ESM.docx]
